# SNPflow: A Lightweight Application for the Processing, Storing and Automatic Quality Checking of Genotyping Assays

**DOI:** 10.1371/journal.pone.0059508

**Published:** 2013-03-19

**Authors:** Hansi Weissensteiner, Margot Haun, Sebastian Schönherr, Mathias Neuner, Lukas Forer, Günther Specht, Anita Kloss-Brandstätter, Florian Kronenberg, Stefan Coassin

**Affiliations:** 1 Division of Genetic Epidemiology, Department of Medical Genetics, Molecular and Clinical Pharmacology, Innsbruck Medical University, Innsbruck, Austria; 2 Department of Database and Information Systems, Institute of Computer Science, University of Innsbruck, Innsbruck, Austria; University of Edinburgh, United Kingdom

## Abstract

Single nucleotide polymorphisms (SNPs) play a prominent role in modern genetics. Current genotyping technologies such as Sequenom iPLEX, ABI TaqMan and KBioscience KASPar made the genotyping of huge SNP sets in large populations straightforward and allow the generation of hundreds of thousands of genotypes even in medium sized labs. While data generation is straightforward, the subsequent data conversion, storage and quality control steps are time-consuming, error-prone and require extensive bioinformatic support. In order to ease this tedious process, we developed SNPflow. SNPflow is a lightweight, intuitive and easily deployable application, which processes genotype data from Sequenom MassARRAY (iPLEX) and ABI 7900HT (TaqMan, KASPar) systems and is extendible to other genotyping methods as well. SNPflow automatically converts the raw output files to ready-to-use genotype lists, calculates all standard quality control values such as call rate, expected and real amount of replicates, minor allele frequency, absolute number of discordant replicates, discordance rate and the p-value of the HWE test, checks the plausibility of the observed genotype frequencies by comparing them to HapMap/1000-Genomes, provides a module for the processing of SNPs, which allow sex determination for DNA quality control purposes and, finally, stores all data in a relational database. SNPflow runs on all common operating systems and comes as both stand-alone version and multi-user version for laboratory-wide use. The software, a user manual, screenshots and a screencast illustrating the main features are available at http://genepi-snpflow.i-med.ac.at.

## Introduction

Single nucleotide polymorphisms (SNPs) are the workhorses of modern genetic-epidemiological research. The replication and deepening of the recent genome-wide association studies (GWAS) [1], [2] demand scalable high-throughput genotyping technologies allowing cost-efficient genotyping in large populations. Two of the most prominent and largely employed techniques are TaqMan probes [3] (commercialized by Life Technologies, Carlsbad, CA, USA) and the Sequenom MassARRAY iPLEX technology [4], [5] (Sequenom Inc., San Diego, CA, USA). TaqMan assays and similar technologies such as molecular beacons [6] or the proprietary KASPar assays (KBioscience, Hoddesdon, UK) harness the fluorescence resonance energy transfer principle (FRET) and provide very robust, single tube genotyping technologies. On the contrary, iPLEX couples single base extension genotyping [7] with mass spectrometric detection and allows the genotyping of up to 40 SNPs per sample in a multiplex assay.

The robustness and simplicity of these technologies allow the generation of hundreds of thousands of genotypes even in medium sized laboratories with limited technical staff and in a short period of time. While the data acquisition is straightforward, the subsequent processing of the raw data represents a major hassle. Researchers face several challenges such as (1) the error-prone manual concatenation of several single, microtiter plate-specific result files, (2) the conversion of platform-specific output formats to ready-to-use genotype lists, (3) the identification of discordant samples and the assessment of the real percentage of samples genotyped twice (i.e. discerning sample names just present twice in the data set from the number of samples, for which two results are actually available), (4) the calculation of quality control (QC) measurements and the assessment of Hardy-Weinberg equilibrium (HWE) violations, (5) a plausibility check by comparing the observed genotype frequencies with the frequencies reported in HapMap [8] and the 1000 Genomes Project [9], (6) the error-free merging of multiple assays into one final result file and, finally, (7) the durable storage of a growing amount of genotypes and QC data. While these issues are solvable for single-plex reactions, they get increasingly error-prone and time-consuming when using multiplex reactions such as iPLEX.

Based on by our experience from several large genetic association studies [10]–[17], we developed SNPflow, a lightweight, free and open-source application for the processing, quality controlling and storage of genotyping results generated on the Sequenom MassARRAY (iPLEX) platform and the Applied Biosystems (ABI) 7900HT RT-PCR system (compatible with FRET-based technologies such as TaqMan assays and KASPar assays). SNPflow fulfills all seven requirements mentioned above and runs on all commonly used web browsers.

## Methods and Software Description

### Implementation

SNPflow comes as either multi-user or stand-alone version. The application has been designed as a Web application based on a client-server architecture using Apache Tomcat (http://tomcat.apache.org/) on server side and JavaScript on client side. Apache Tomcat is an open-source, securely encoded HTTPS web server and runs the core of SNPflow, which is implemented in the Java programming language (http://www.java.com). The user interface on client side is implemented with the Ext-JS JavaScript framework (http://www.sencha.com/products/extjs), which allows the generation of a desktop-like interface inside a web browser.

The communication between server and client is handled by servlets. The standard application flow is that the client, i.e. the web browser, sends a request to the server where it is received and processed by request-specific java servlets (e.g. DataUploadServlet for uploading a file or UserServlet for editing or adding a user). On client side data is exchanged via JavaScript Object Notation (JSON, http://www.json.org/) objects. A freely available relational database (MySQL for the multi-user version (http://www.mysql.com), H2 (http://www.h2database.com) for the standalone version) is used to store all user and study-specific information on server side. HapMap genotype information release 27 is accessed via the BioMart web service of the HapMap project [18], [19] called HapMart, transferred to SNPflow and stored in the database to minimize the network traffic. HapMap release 28 is still not available in HapMart but will be implemented in a next version of SNPflow as soon as release 28 becomes accessible. The 1000 Genomes Project data is accessed via the Ensembl ’s database [20]. We were not able to use Ensembl’s BioMart web service [21] since that provides only the minor allele frequency (MAF) and the minor allele count (MAC) pooled (“global”) for all populations and does not provide this information for each subpopulation.

The communication between the server and the database is executed by the Java Database Connectivity API (JDBC) driver as well as the Hibernate framework (http://www.hibernate.org). Hibernate is a powerful object-relational mapping tool for persistence and query services. The passwords of the different users are stored in the database securely encoded by MD5 hash. For file generation and data export the JavaCSV (http://sourceforge.net/projects/javacsv/) library for delimiter-separated files and the JExcelApi library (http://jexcelapi.sourceforge.net/) for Microsoft Excel files have been utilized. The JExcelApi allows the definition of cell formats, to avoid wrong interpretation e.g. auto conversion errors of data values (numbers as dates or vice versa) in Excel.

### Standalone Version

Besides the multi-user version, we also provide a standalone version, for users who do not have the system rights to install new software or do not have the in-house server infrastructure to set up MySQL and Apache Tomcat. The standalone version of SNPflow is delivered with an integrated relational database (H2) and an embedded Apache Tomcat 7 server and only requires Java to be installed separately. This eliminates the server set-up and allows starting SNPflow on every local computer system with just a single click. The standalone version of SNPflow provides a very convenient way to test the application without the need to install a database and a web server. It presents, however, the shortcoming that data is stored at the user’s local workstation and can therefore not always be accessed by other users. For multi-user capability the installation of the standalone version is not required.

SNPflow is platform-independent, has been tested on Windows, Linux and MacOS environments and runs on the most common browsers (tested on Mozilla Firefox 3.6–18, Microsoft Internet Explorer 8, Google Chrome 24, Safari 6, Opera 12).

### Compatible Input Formats

SNPflow is able to deal with results coming both from the Sequenom TyperAnalyzer 4.0 software and the ABI SDS software version 2.2.2, since these two platforms are present in our laboratory and we were thus able to implement SNPflow respectively. As long as the output format does not change, other versions of these software packages might work too, but should be thoroughly tested (examples of working outputs files are provided on the website). We also welcome collaborations to implement and intensively test other software versions or output formats in the future and encourage programming-experienced users to take advantage of SNPflow’s open-source license to modify it accordingly to their needs.

## Results

### User Interface

**Figure 1:**
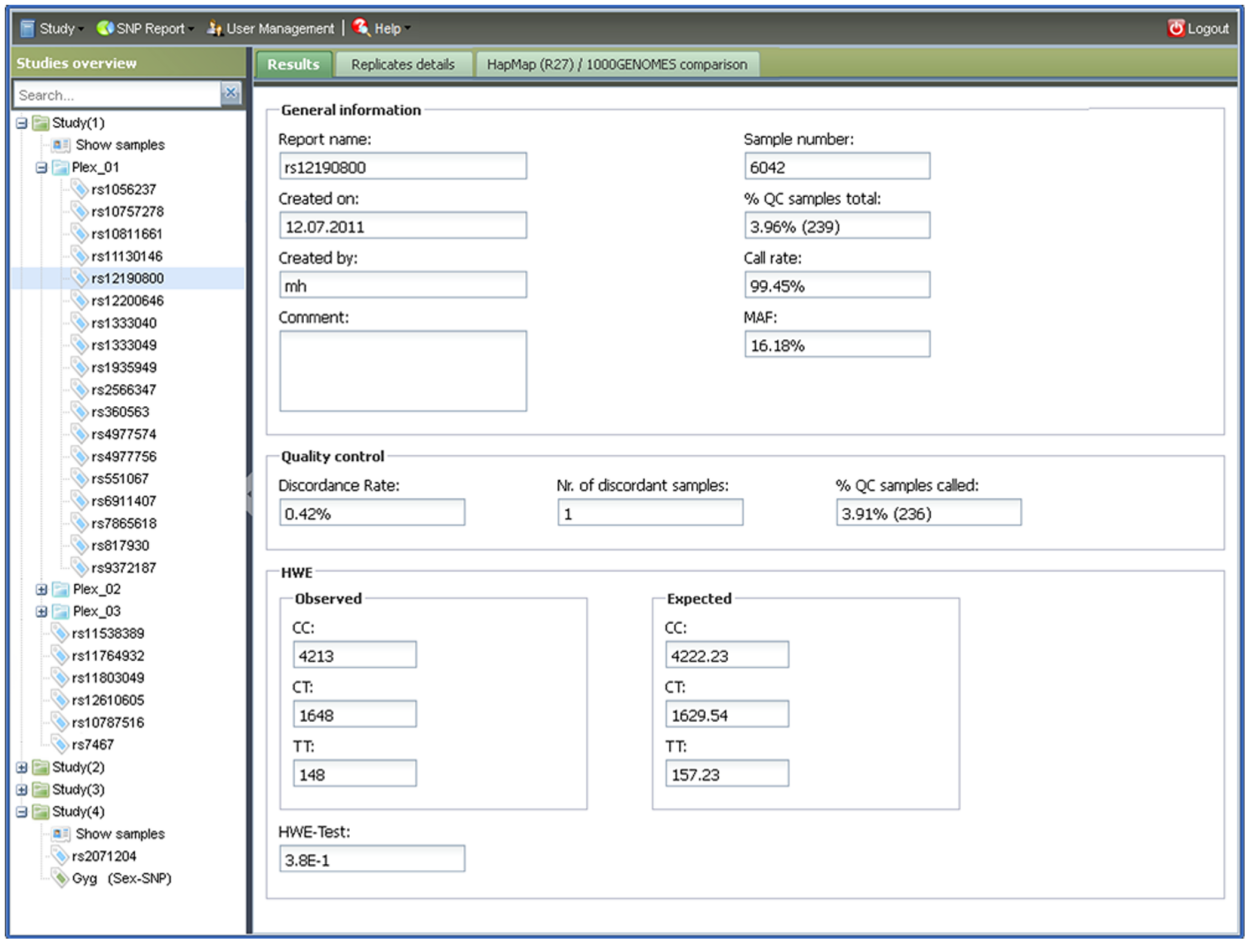
Main interface of SNPflow. This figure shows the main interface of SNPflow. The left panel illustrates the different studies with the respective SNP reports. iPLEX Assays are grouped into subfolders, (e.g. “Plex1”) while TaqMan results are reported as single documents (e.g. “rs2071204” in “Study (4)”). Specific SNP results can be retrieved with the search field in the study overview. The right panel contains three tabs showing the QC data, detailed replicates analysis and the comparison with HapMap release 27 and the 1000 Genomes Project phase 1 (see also figure 4). The tool bar with the different options is located on the top of the window.

SNPflow has been designed to store genotyping results in a study-oriented structure. The SNPflow interface consists of two main panels (Figure 1): The left panel displays all study populations in a tree-like structure, which allows navigating through the data by simple mouse clicks. The sub-tree of each study population includes the sample identifier (ID) list (tree node “show samples”) and the genotyping results. Sequenom MassARRAY iPLEX assays are displayed as sub folders while results coming from the ABI 7900HT are shown as single result files. Specific results can be retrieved quickly by using the search field on the top of the left panel. A double-click on a SNP opens in the right panel the “Results tab” with the QC values grouped by “General information”, “Quality control”, “HWE” and “HWE-Test”. Two additional tabs (“Replicate details”, “HapMap/1000-Genomes comparison”) provide detailed information about discordant replicates, a list of replicates that were called only once and a comparison between the allele frequencies observed in the data and the frequencies reported in HapMap rel27 and the 1000 Genomes Project phase 1, respectively.

The toolbar on top of the panels provides four buttons: “Study”, “SNP Report”, “User Management” and “Help”.

The button “Study” provides tools for adding, editing, activating and deactivating studies (i.e. hiding studies, which are no longer used) and for starting the processing of genotyping data (for details please see the section Workflow).

The “SNP Report” button allows exporting or deleting selected results. Results can be exported to Excel worksheets.

The third button “User Management” gives access to the user account settings. Three different user roles are provided: Admin, Lab and Analyst. The role management assigns full privileges to the Admin role, reduced privileges to the Lab role and fewest rights to the Analyst role. “Lab” users are allowed to both upload and delete SNP assays, but have no access to study data and user management, while “Analysts” are only allowed to view and export already generated data. Due to the central role of the study data, only “Admins” are allowed to modify study-related data such as the sample IDs or number of expected replicates. The user creation is currently a manual process. Since the user management is kept very simple (username, password, role) and the amount of active users is manageable, the effort is small to add or edit users.

The “Help” button includes the link to the user manual and to the SNPflow website, where you can find the example files for testing the program.

Most functionality from the toolbar can be executed also by using the right mouse button on a specific node in the navigation tree.

### Workflow

#### 1. Upload of the study data

**Figure 2:**
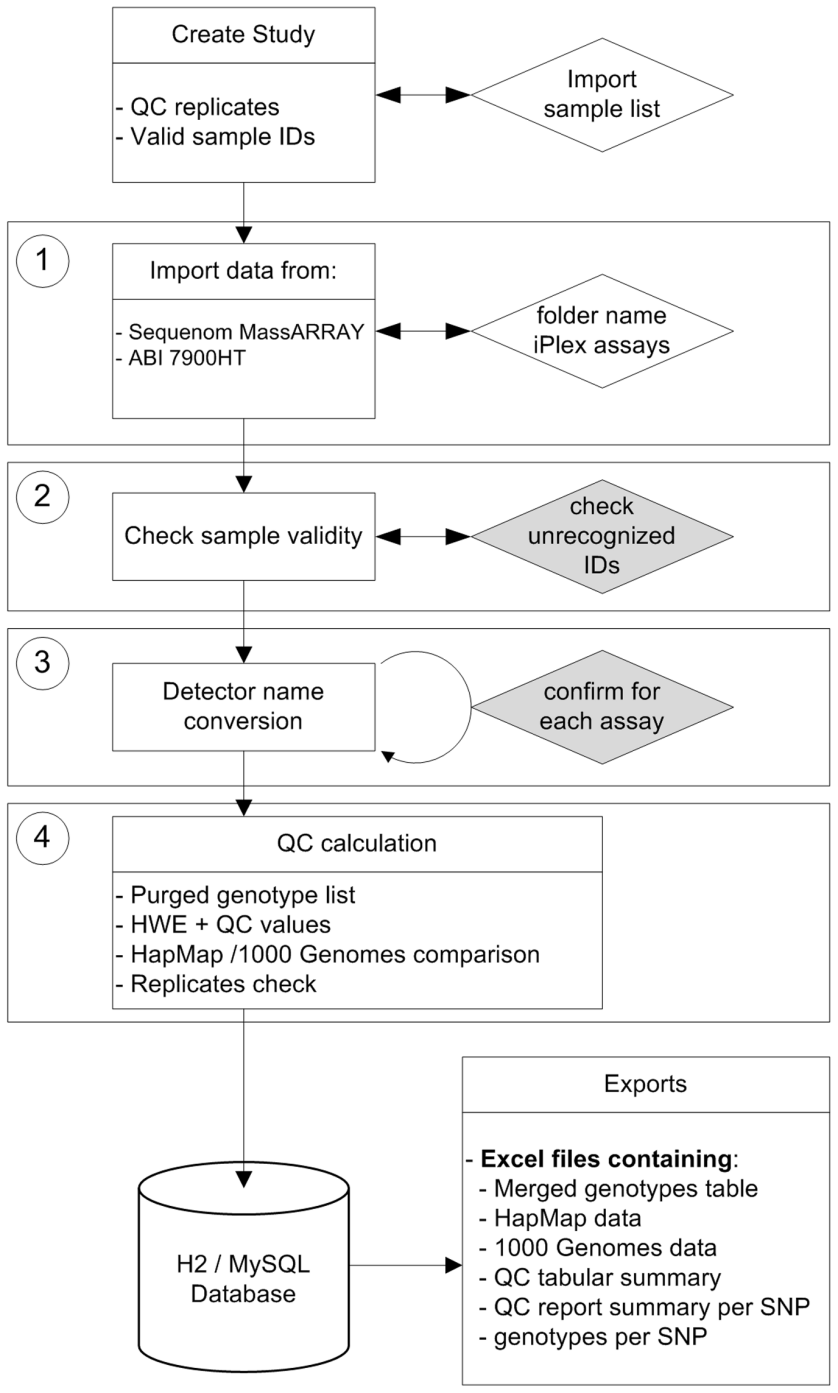
Flow chart of the main data processing workflow. User actions are depicted as diamonds. The two shaded diamonds represent the two major data checkpoints as described in the subsection “Processing of genotyping data” in the “Workflow” section of the main text. The numbers of the four boxed steps correspond to the steps of the four SNPflow data analysis wizard (see also figure 3). For a detailed description of this flow chart, please see the section “Workflow” in the manuscript.

The SNPflow workflow (Figure 2) starts with the definition of the study properties by setting the study name, uploading the sample IDs and providing the number of replicates on the plates. The uploaded sample IDs are used to identify all wells containing identifiers that are not denoting study individuals, such as non-template controls (e.g “NTC”) or positive controls (e.g. “PC”). This set-up allows the flexible handling of all kinds of definitions for positive or negative controls. Moreover, it allows the inclusion of sample IDs in the final output, which are part of the study, but that were not present in any of the uploaded result files. This can happen, for example, when a certain well is excluded from the allelic discrimination scan on the 7900HT system by not assigning any SNP assay information to the well. In this case, the information for this well is not present in any of the result files, thus distorting QC calculations and result files.

#### 2. Processing of genotyping data

**Figure 3:**
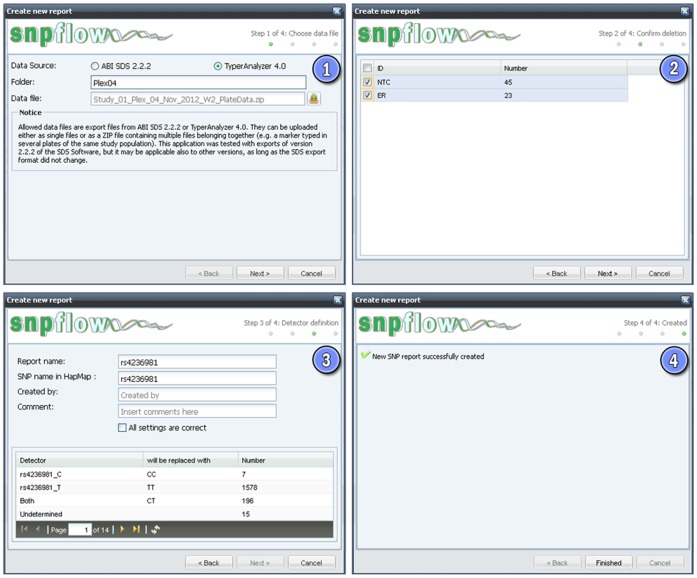
The SNPflow data analysis wizard. This figure shows the 4 steps of the SNPflow data analysis wizard. In brief, the first step allows the uploading of either SDS files (ABI 7900HT) or TyperAnalyzer 4.0 (MassARRAY) files. For TyperAnalyzer files, a second input field asks for the name of the result group under which results shall be grouped. The second step checks the validity of the IDs found in the uploaded and reports IDs, which are not found in the study definition and shall thus be deleted. Step 3 converts the found genotype designations to standard genotypes and allows entering the name of the result, the operator name, an optional comment and the rs-number of the SNP in HapMap and the 1000 Genomes Project. This is useful in case that a SNP ID changed between the current dbSNP release and the dbSNP release used in HapMap, as happened for some SNPs. For convenience, the result name is automatically pre-set as HapMap lookup. In multiplex assays, pagination allows navigating through the single SNPs. For quality reasons a manual confirmation of each conversion is required (Checkbox “All settings are correct”). Finally, step 4 confirms the successful analysis and brings the user to the report. Exemplary abbreviation used in this figure: NTC, Non-template control; ER, annulled samples (“error”), i.e. DNA samples present on the plates, but known to be flawed. The IDs of these samples were thus replaced with “ER” in order to avoid data collection for these samples.

The processing of the genotyping results is organized in an intuitive and fast four step wizard (Figure 3). SNPflow contains two check-points, which require a manual confirmation by the user. This provides high confidence in SNPflow’s results and ensures compatibility with most particularities of the user data sets, since the checkpoints provide a direct control on the major deletion and conversion steps.

The wizard starts with the upload of the output files from the data analysis software (SDS or TyperAnalyzer 4.0). The output files can be uploaded either as a single file or, in the case that a study spans several 384 well microplates, as a zip archive (step 1). SNPflow then compares the IDs found in the merged data set with the IDs stored in SNPflow’s study definitions and proposes unrecognized sample IDs for removal (step 2; first data checkpoint). Subsequently, SNPflow detects the genotype designations used in the data files and suggests the corresponding genotypes for the final result file. For security and quality reasons, the suggested genotype conversions have to be approved manually for each SNP assay (step 3; second data checkpoint). Please note that, since the detector definitions in the SDS software are arbitrary, we assume that the detector designations adhere to the following pattern: “<arbitrary detector name>_<allele>” (e.g. “rs328_G” or “LPL-rs328_G”). SNPflow works also with any other kind of detector definitions, but the automatic genotype detection might require manual editing. This issue is not present with MassARRAY files, as these are better standardized than SDS output files. Finally, all data is stored in the database and displayed in a QC report (step 4).

#### 3. The QC report

**Figure 4:**
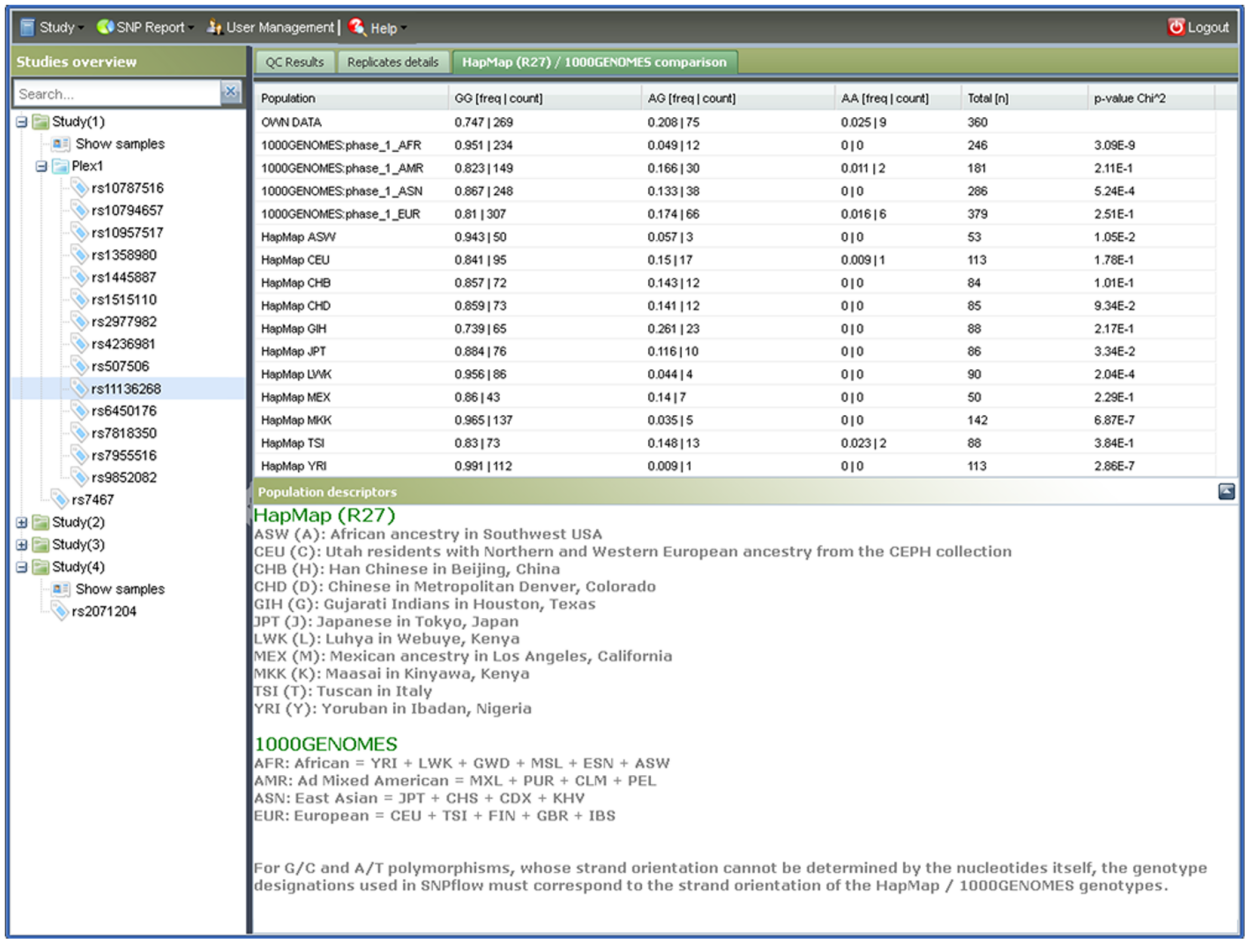
SNPflow’s HapMap/1000Genomes comparison panel. This figure exemplifies the HapMap/1000Genomes comparison. Both, frequencies and absolute genotype numbers are reported for each population. A Pearson chi square test checks the concordance with the user’s data. The user can look up the most appropriate HapMap population as well as the super populations from the 1000 Genomes Project and perform an additional plausibility check of the generated genotypes.

SNPflow’s QC report contains both descriptive data and standard QC values (Figure 1, right part of the screen). For each SNP assay SNPflow provides creation date, operator name, comments, call rate, expected (i.e. the total number of replicates in the experiment) and real (i.e. the number of replicates, which actually produced usable data) amount of replicates, minor allele frequency, absolute number of discordant replicates, discordance rate and the p-value of the HWE test. Furthermore, SNPflow lists the IDs and positions of discordant replicates and of replicates typed only once. Finally, SNPflow checks whether a SNP has been typed by HapMap (release 27, containing data from all three HapMap phases [8], [22], [23]) and the 1000- Genome Project (phase 1) and, if found, automatically retrieves the reported frequency data for all HapMap populations as well as the super populations from the 1000 Genomes project. The statistical significance of differences between the allele frequencies observed in the user data set and the frequencies expected from the HapMap/1000Genomes data is assessed by a Pearson Chi-squared test (Figure 4). When compared to the most appropriate ethnicities, this allows the immediate detection of strong deviations in the expected frequency distribution, as they might arise, for example, by mixing up of probe identifiers.

#### 4. Export of data

The finished genotype lists and the QC values are exported in single output files and merged Microsoft Excel files containing data from several independent SNP assays within an study (merged genotype list and merged QC report for all selected SNPs) providing an immediate overview of the QC values of all selected SNPs. This simplifies the handling and merging of results from large genotyping projects with several single assays or employing both realtime-PCR and MassARRAY technology. In the Excel QC-report files values exceeding certain thresholds (e.g. HWE Test <0.05) are automatically highlighted in order to enhance the readability of the output files (for details see the SNPflow User Manual available on the project website). Moreover, the user can select which HapMap/1000Genomes populations have to exported, thus avoiding the useless export of incomparable populations.

### Processing of SNP Markers of Sex Determination

Single nucleotide differences in genes present on both the X and the Y chromosome ((amelogenin (AMELX, AMELY), glycogenin 2 (GYG2), ZFX, ZFY)) allow sex determination by SNP genotyping [24]. Since such SNP markers are commonly used in genetic epidemiology to check study populations for sample mix-up (as done e.g. in [25]), SNPflow provides an additional analysis module to check the genotyped samples for sex concordance. Any SNP stored in SNPflow can be selected by mouse right-click and set as “sex determination SNP”. This opens a dialog box, where the user can upload a list of sample IDs together with the expected sex phenotype of each sample and assign the genotypes to a specific sex phenotype. The sex coding in the uploaded file can be selected freely and defined in the dialog box. SNPflow then compares the genotypic sex with the uploaded phenotypic file and reports discordances in a separate result tab.

Sex data is only used for comparison and only discordances are stored in the database in order to avoid the storing of sensitive phenotypic data together with genotypic data.

## Discussion

### Comparison to Other Software Solutions

While modern genotyping technologies allow unprecedented throughput and cost-efficiency even in medium-sized labs, the storing, processing and critical evaluation (i.e. QC check) of the genotyping data is still time-consuming and error-prone. Large genotyping facilities often use ad-hoc scripts written e.g. in R or Visual Basic, but hardly any comprehensive solution exists for medium sized labs with limited bioinformatic support. Moreover, these solutions are often improvised, do not use state-of-the-art informatics or are limited to a specific computer platform.

On the contrary, SNPflow is a lightweight application, which eases the processing and storing of genotypes generated on ABI 7900HT RT-PCR (compatible with most FRET-based genotyping technologies such as TaqMan or KASPar) and Sequenom MassARRAY systems. Our tool can be easily installed directly at the users’ institution server (multi-user version) or PC workstation (stand-alone version) and is accessed via a common web browser.

Some other groups have developed bioinformatic solutions addressing at least some of the issues mentioned in the Introduction section [26]–[37]. These range from customary Visual Basic scripts for the reformatting of Microsoft Excel files (T.I.M.S. [30]) to comprehensive laboratory information management systems (LIMS) (SNPator [34], IGS [37]). We would like to briefly discuss here four solutions, which we regard to be partially comparable to SNPflow: Genotool [32], T.I.M.S. [30], SNPator [34] and IGS [37]. Table 1 summarizes all relevant features compared to SNPflow.

**Table 1:** Comparison to related software.

	Genotool*	T.I.M.S.	SNPator	IGS*	SNPflow
Last Update	2001	2005	2008	2006	2013
Operating System	MS Windows	MS Windows	Linux	MS Windows Server	MS Windows, Linux, Mac OS X
Programming Language	VB	VBA	PHP	C#, VB.Net, ASP.Net	Java
Database	MS SQL Server#		MySQL	MS SQL Server#	MySQL/H2
Additional Requirements		MS Excel#		IIS 6.0#	
Software Architecture	Client/Server	Excel Add-In	Web Application	Web Application	Web Application and Stand-alone
Open source	no	yes	no	yes	yes
Data Hosting	In-house	In-house	Outsourced	In-house	In-house
User-Management†	no	no	yes§	yes	yes
**Quality Checks**					
HWE	no	yes	yes	yes	yes
HapMap comparison	no	no	yes	no	yes
1000-Genomes comparison	no	no	no	no	yes
Sex determination SNP	no	no	no	no	yes
**Supported methods**					
ABI Taqman file	yes	yes	yes	yes	yes
TyperAnalyzer plate data (Sequenom)	no	no	yes	yes	yes
Illumina	no	no	yes	yes	*On request*

* Evaluation based on publication.

# Requires license.

† User management means the definition of user roles in the sense of rights for e.g. administrators, laboratory analysts or data analysts.

§ Should be available in the ‘pre-packaged’ version of SNPator which is, however, not publically available.

Genotool enables the management of genotypes, phenotypes and pedigree data from TaqMan genotyping. Genotypes can be imported directly from TaqMan data and checked for Mendelian inheritance errors. Unfortunately, Genotool relies on the commercial database server MS SQL 7 as well as outdated Windows versions (95/98/NT). Therefore we could not test the software.

The T.I.M.S. (TaqMan Information Management System) package is a very extensive collection of Visual Basic scripts in Microsoft Excel, which was designed to improve the processing of input and result files from the 7900HT system. T.I.M.S. merges single result files, calculates allele frequencies and HWE violations and assesses concordance of duplicated samples. The results can be exported into several output formats for further analysis with other tools. T.I.M.S. lacks, however, an underlying database and the data is exported into an Excel file. Without a well-organized central storage of the result files, this harbors the risk of losing data files, especially in laboratories genotyping hundreds and thousands of SNPs and with pronounced staff turnover as it is often the case in the academic sector. Moreover, T.I.M.S. requires adhering to a specific pattern of detector and marker designations. Albeit also SNPflow works best with a specific format of the detector settings in the 7900HT system (see the section “Workflow”), the graphical interface of SNPflow allows the use of virtually any kind of designations and the user can easily check and edit the designations and conversions proposed by SNPflow. Accordingly, T.I.M.S. requires marking replicates with the prefix “QC-“, while SNPflow detects replicates automatically.

SNPator is actually the customer interface of the National Spanish Genotyping Center [34]. It is a very powerful, web-based LIMS with the possibility to manage samples, store phenotypic data, process genotypes from different systems (Illumina, Sequenom, SNPlex) and save marker-related information in a centralized MySQL database. It furthermore allows the calculation of numerous quality control values such as HWE and also provides data analysis modules for performing elementary association tests. The broad range of features available in SNPator is clearly very useful for users without strong knowledge in genetic epidemiology, but might discourage expert users, who have an already established data analysis pipeline and are just searching for a slender tool for the automatic processing of genotyping data. Most important, SNPator is accessible only via the web. Since many laboratories might not be willing to upload sensitive data to public servers, in our opinion a purely web-based interface could limit the acceptance of a bioinformatic application in the community. We therefore refrained from running SNPflow as an application on our own servers and making it available to the community only via an internet-interface in favor of an application running locally at the users’ place to avoid data security issues [38].

Similarly, IGS is the LIMS system of the Wellcome Trust Centre for Human Genetics (Oxford, UK) [37] and provides features for the administration of sample IDs, phenotypic data, microplates, marker-related information and genotyping data. The processing of raw data from genotyping systems is only a minor feature of the whole application. Both IGS and SNPator are thus only partially comparable to SNPflow, since SNPflow has been deliberately designed as a lightweight, intuitive and easily deployable application for the data processing in the wet lab, without addressing data analysis or the administration of phenotypic data. For users looking also for a solution for the management of study populations and phenotype data, our workgroup developed eCOMPAGT [39], [40], which perfectly complements SNPflow and allows strict separation of genotypic and phenotypic data, as might be required by some institutions.

Furthermore two solutions for GWAS and SNP linkage analysis where taken into consideration for comparison with SNPflow, namely PLINK [41] and GATK [42]. As stated on the project site (http://pngu.mgh.harvard.edu/~purcell/plink/), PLINK’s focus is purely on analysis of genotype/phenotype data, with no support prior to these steps (e.g.generating genotype calls from raw data) [41]. This support, however, is exactly the focus of SNPflow, acting a stage earlier than PLINK. Therefore an export to PED and MAP files is planned in a future release of SNPflow to extend the pipeline for further data analysis with PLINK. In contrast GATK’s focus lies on variant discovery and genotyping analyse of next-generation resequencing data and is only intended to process SAM/BAM files [42]. The three softwares are therefore not directly comparable to each other.

### Limitations of SNPflow

We would like to briefly discuss also two potential limitations of SNPflow. First, the current SNPflow release only guarantees to handle studies with population sizes up to 20,000 samples in a feasible time (<10 minutes). For larger studies the Java Heap Space has to be increased and the calculation of SNP quality values requires linearly more time.

Second, SNPflow focuses on the 7900HT RT-PCR system and the MassARRAY MALDI-TOF system, two widely-used systems for targeted SNP genotyping. We focused on these two systems because both are available in our laboratory and we are therefore able to ensure thorough and well-founded testing of SNPflow. Indeed, by allowing the use of iPLEX, TaqMan and KASPar chemistry, the 7900HT RT-PCR and the MassARRAY systems together meet the genotyping requirements of most current association studies. For example, 17 out of 19 de-novo replication studies used in Heid et al. [43], 18 of 19 replication studies used in Speliotes et al. [44] and 17 of 18 replication studies in Voight et al [45] employed one of these three technologies. We thus believe that SNPflow will be helpful for a large part of the genetic community. We are, however, aware that some users might use other platforms and welcome collaborations to implement additional replication genotyping platforms in future.

### Strengths of SNPflow

Despite these two limitations, SNPflow presents major advantages for the processing of genotyping data and helps to avoid important pitfalls in the manual or semi-automated processing of SNP data. For instance, the automated processing of the SDS and Typer 4.0 export files avoids the highly error-prone and time-consuming merging and conversion of single files. This is mostly done by MS Excel, which present several pitfalls like sorting of only one column instead of all columns or wrong “replace all” settings.

The automatic comparison with HapMap and the 1000 Genomes Project provides a powerful way to check the plausibility and validity of SNP data, which successfully passed the other QC values. This can be easily the case, when, for example, the designations of the detectors are accidentally inverted. In this case, the results would still fulfill requirements of HWE concordance and present proper call rates. Conversely, sample mix-up can be detected by the automatic sex concordance check. Taken together, SNPflow allows the detection of most kinds of error still in the wet-lab and helps to tackle genotyping problems directly where they originate. This avoids releasing erroneous data to the data analysts and prevents that genotyping errors are not detected until implausible association study results or incongruences with external data arise.

Finally, the possibility to export several SNP results into already merged genotype lists avoids time-consuming and highly error-prone merging of single results. Once exported, the genotype file can be directly forwarded to data analysts and cooperation partners without further handling and editing and together with a comprehensive QC report. To the best of our knowledge, only the overly comprehensive LIMS solutions SNPator is able to directly handle MassARRAY data and no lightweight solution for the processing of MassARRAY data similar to SNPflow exists.

### Conclusion

With SNPflow we offer the community a lightweight application for the processing of genotyping data from two major genotyping platforms: the ABI 7900HT system and the Sequenom MassARRAY system. SNPflow provides an intuitive workflow to perform quality control of QC data and ensures reliable and ordered data storage in a relational database. SNPflow can especially support medium sized labs without strong bioinformatic support and optimally complement existing solutions.

The SNPflow software, a user manual, additional screenshots and a screencast showing the use of SNPflow are available at http://genepi-snpflow.i-med.ac.at.
